# Varied Experiences in a Surgical Ward

**Published:** 1914-04-11

**Authors:** 


### HOSPITALS FROM THE PATIENTS' POINT OF VIEW.

(Contributions to this Section are Invited.)

### Varied Experiences in a Surgical Ward.

The patient whose impressions of hospital sojourn are recorded below came into the surgical ward of a general London hospital for an abdominal operation. His opinions are of considerable value since he had some previous experience of hospital treatment as an in-patient in a large American hospital at New York and also of treatment in a private nursing clinic on the Continent.

"The condition for which I had to enter hospital was an old trouble from which I had experienced considerable inconvenience, if not actual danger, for some time past. Indeed it had necessitated surgical treatment on two occasions, and when I was told that a third operation was advisable I endeavoured to do what I had done before—namely, to enter a private nursing home. I found, however, that the charges were exorbitant, and I was told, in addition, that I would fare far better if I entered a general ward. While I could pay a moderate fee, I could not afford the expenses entailed by a prolonged stay in a private nursing home, and accordingly—I confess somewhat shamefacedly—I accepted my kind doctor's offer to get me into a surgical ward. Let me say at once that I have not regretted that step. What I wish to say here is in the nature of hints that I hope may prove helpful—not at all in the nature of carping criticism that postulates ungratefulness on my part or a forgetfulness of the kindness and general consideration with which I was treated while in hospital."

### A Plea for the American System.

"It appears to me that the hospitals in this country make a great mistake in not dividing their patients into paying and non-paying groups as is done in America. I feel sure that many patients are chary of taking a bed in a general ward simply because they feel, as I felt, that they are utilising something that was meant, primarily, for people who could not afford to pay a penny towards their treatment. Now some of us can afford to pay a small amount, which, although it may not cover the cost of treatment, serves at least two purposes: it obviates the patient from thinking that he is living exclusively upon charity and enables him, in however slight a measure, to give something in return. A small paying ward attached to every hospital would be a great convenience and would go far to discourage the frequent abuse of the hospitals by patients who can afford to pay something. From what I gathered there were several patients in the ward who were situated as I was; they could pay a small amount, but could not afford the large private operating fees and the heavy extras entailed by treatment in a nursing home, and so they did as I did, gratefully accepted their doctor's offer and came into hospital.

"I need scarcely say that no difference whatever was made between such patients and really charity patients in the hospital I was in. So far as the actual nursing was concerned, I think I was better and more comfortably nursed here than in the American hospital, though perhaps not so skilfully as in the German clinic, where the surgeon, however, supervised everything, even to the changing of the draw-sheet."

### Early Morning Disturbance and Unfortunate Visiting.

"My sole complaint was the early morning disturbance, which was commenced at what seems to me an entirely unnecessarily early hour, and the kindly meant but undoubtedly distracting attention of several good ladies who regularly 'visited' the ward and supplied us with old-fashioned tracts. Alone and practically friendless as I was, I would have welcomed any visitor who could talk with me on an interesting topic; but I found these visitors and I had very little in common, and our interviews were, I believe, mutually unsatisfactory.

"A trifling point perhaps, but one which caused me discomfort, was the way liquid food was administered. I found the drinking-cup an abomination when lying flat in bed or on my side; in the former position it nearly always necessitated the help of a nurse. In the German clinic I was given an ordinary glass or tumbler, which was placed on the bed-locker close to my side, and I drank its contents through a flexible tube—a far more convenient and pleasant way of absorbing liquids for a sick person.

"The food I found quite well cooked and tasty, and, on the whole, admirably served; the only discomfort was eating it on the bed, since no bed-table was provided."

### Call-bell and Bedding Suggestions.

"An undoubted defect was the absence of any bed-call for the nurse. There was no way of attracting her attention when she was at the other end of the ward than by calling out, a method which fixed attention on the poor patient and necessitated some exertion on his part. A bed button-bell or a light signal such as is used in American hospitals seems a great improvement on this method. My own bed I found at first exceedingly uncomfortable, since the mattress was unevenly stuffed. When I complained this was at once altered, but surely it should have been seen to before a patient was put into the bed. The blankets supplied were of decidedly inferior quality, as I found out when my hot-water bottles were discontinued. Perhaps I felt this change more acutely since the ward was not overheated in the American fashion. The head pillow-cases were open, and frequently got disarranged so that some of the feathers worked out of the pillow-cover itself and irritated my face. I suggest that such cases should be fastened with safety-pins or buttons, as I believe is done in some hospitals. That would add greatly to the patient's comfort.

"I am not particularly 'squeamish' or sensitive, and I had no objection whatever to students' demonstrations. But it did seem to me that the teaching surgeon, when demonstrating on a particular case, might refrain from taking the whole ward into his confidence. Shortly after my operation, when I had begun to take an interest in my surroundings, the bed next to mine was visited by a posse of students in charge of a demonstrator, and I had to listen for half an hour to the undoubtedly interesting possibilities in the way of complications after an abdominal operation. Some of these were a revelation to me, and I confess that for some days I anxiously studied my temperature chart, counted my pulse, and took deep breaths to assure myself that general peritonitis was not approaching me. Such a discussion may just as well be indulged in at the far end of the ward to refrain from harrowing the patient's feelings and those of his immediate neighbours. When one has just undergone an abdominal operation and been told that everything is all right, it comes as a shock to hear that such things as 'delayed chloroform poisoning,' peritonitis, meteorism, obstruction, ulceration, and embolism are things which might still happen to one within the next few days and be generally accepted as by no means out-of-the-way occurrences!

"The ward in which I was is a very draughty one, and the circulation of air is particularly free. A screen by the door and one or two arranged about the windows to deflect the draught currents would make it much more comfortable, but my suggestion to that effect was not taken kindly by the ward Sister.

"One final criticism—again most diffidently offered. Is it necessary to starve a patient before an operation in that absolute degree which I experienced? I had no supper the night before my operation; this I afterwards found was a 'slight oversight' on the part of someone. My breakfast consisted of weak tea, so that for practically twenty-four hours before I was operated upon I had no food. In my own case perhaps this was necessary—I offer no opinion on this point—but it appeared to be the rule in the ward. Both in America and in Germany I had been given a fairly nourishing breakfast some six hours before the operation, and I felt all the better for it."

